# Ultrasensitive Terahertz Biosensors Based on Fano Resonance of a Graphene/Waveguide Hybrid Structure

**DOI:** 10.3390/s17081924

**Published:** 2017-08-21

**Authors:** Banxian Ruan, Jun Guo, Leiming Wu, Jiaqi Zhu, Qi You, Xiaoyu Dai, Yuanjiang Xiang

**Affiliations:** SZU-NUS Collaborative Innovation Center for Optoelectronic Science & Technology, Key Laboratory of Optoelectronic Devices and Systems of Ministry of Education and Guangdong Province, College of Optoelectronic Engineering, Shenzhen University, Shenzhen 518060, China; 2161190229@email.szu.edu.cn (B.R.); guojun@szu.edu.cn (J.G.); wlm40588@163.com (L.W.); 2161190233@email.szu.edu.cn (J.Z.); 2161190234@email.szu.edu.cn (Q.Y.); xiaoyudai@126.com (X.D.)

**Keywords:** biosensor, Fano resonances, surface plasmon polaritons, graphene

## Abstract

Graphene terahertz (THz) surface plasmons provide hope for developing functional devices in the THz frequency. By coupling graphene surface plasmon polaritons (SPPs) and a planar waveguide (PWG) mode, Fano resonances are demonstrated to realize an ultrasensitive terahertz biosensor. By analyzing the dispersion relation of graphene SPPs and PWG, the tunable Fano resonances in the terahertz frequency are discussed. It is found that the asymmetric lineshape of Fano resonances can be manipulated by changing the Fermi level of graphene, and the influence of the thickness of coupling layer and air layer in sandwich structure on the Fano resonances is also discussed in detail. We then apply the proposed Fano resonance to realize the ultrasensitive terahertz biosensors, it is shown that the highest sensitivities of 3260 RIU^−1^ are realized. Our result is two orders of a conventional surface plasmon resonance sensor. Furthermore, we find that when sensing medium is in the vicinity of water in THz, the sensitivity increases with increasing refractive index of the sensing medium.

## 1. Introduction

Optical sensors based on surface plasmon polaritons (SPPs) at noble metal (such as gold and silver) surfaces [1,2] have been widely studied since Otto and Kretschmann excited SPPs with attenuated total reflection (ATR) [3,4]. Actually, a SPP is a kind of surface wave that is excited by a collective free electron oscillation on the metal surface, induced by the external electromagnetic field. When the wave vector mismatch between an SPP and the incident transverse magnetic (TM) polarized light is compensated, a reflection dip always appears in the reflectance-angle/wavelength spectrum implying the existence of SPP. These sensors are found to be extremely sensitive to the changes in the refractive index of any dielectric attached to the metal surface, which make them have extensive applications in areas such as medical diagnosis [5] environmental monitoring [6] and the analysis of biomolecular interactions [7].

When the sensors are applied to biological detection, in contrast with traditional biosensors in the visible frequency, terahertz frequency biosensors are more suitable because their low-photon energy characteristics can excite the collective oscillatory mode of biomolecules and enhance the sensitivity of the biosensing molecules [8]. In the recent decades, the rapid development of ultra-fast laser technology and semiconductor technology have provided a stable and reliable excitation light source for THz pulses [9,10], attracting a lot of interest in THz applications, especially in the detection aspect. Traditional metal SPPs suffer from their large intrinsic losses, no tunability and limited working spectrum (only from the visible to near-IR wavelengths), but graphene can make up for these problems. Gan et al. have given a detailed analysis via ATR of surface plasmon excitation at terahertz frequency with highly doped graphene sheets [11,12]. Compared with conventional plasmonic materials such as noble metals, its THz response can be dynamically tuned by electrostatic gating. In addition, the electromagnetic field of the graphene THz plasmonic materials shows a better spatial confinement [11,12,13,14], which makes them extremely attractive for enhanced light-matter interactions. Exceptional optical and electrical properties of graphene give it good application prospects.

Terahertz sensors based on graphene SPPs have been investigated by several authors [13,14,15,16,17,18]. Gan et al. reported Otto geometry for SPP excitation in terahertz frequencies using graphene [13], Srivastava et al. proposed a gas sensor based on Gan et al.’s work and just replaced the air layer with a dielectric spacer layer [14]. Singh et al. demonstrated sensors with terahertz metamaterial [15,16,17,18]. Accurate detection of microbiological and biological substances, and the pursuit of sensors with higher sensibility, have always been a major goal of bioanalytical technology [3]. Fano resonances applied in sensors is one of the most effective ways to improve the accuracy. Fano resonance, first proposed by Ugo Fano to describe the asymmetric autoionization spectra of He atoms [19], has also been studied in plasmonics [20,21]. Hayashi et al. proposed a Fano-based sensor via the coupling between metal-excited SPP and PWG mode [22]. It improved the sensitivity, but was limited to only visible frequencies. Guo et al. proposed a structure composed of few layer graphene (FLG) and PWG to realize Fano resonance in mid-infrared wavelengths [23]. Singh et al. realized Fano resonance in THz wavelengths by using metamaterials/metasurfaces [24,25,26,27]. Unlike natural materials like graphene that respond in the THz frequency range because of their chemical constituents, the optical behavior of metamaterials depends on interactions in the lattice array.

Here, we have proposed a THz biosensor based on the Fano resonance. The coupling between a broad resonance supported by FLG SPP and a narrow resonance supported by PWG mode leads to a sharp Fano resonance. We believe that the sharp line shapes can have a good potential as highly sensitive biosensors in the THz frequency range.

## 2. Theoretical Models and Methods

**Figure 1:**
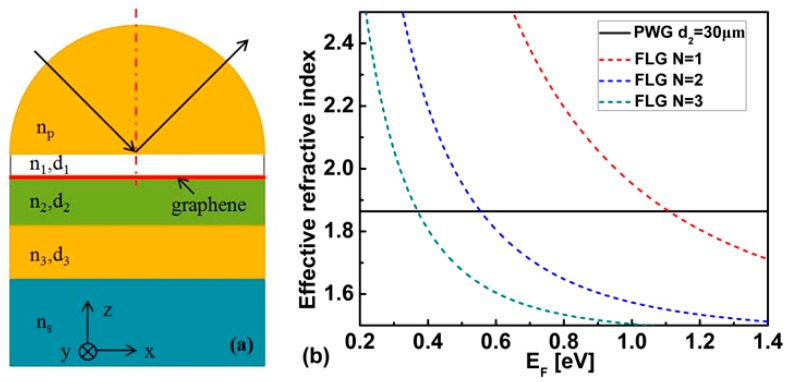
(**a**) Schematic diagram of the proposed biosensor based on Fano resonance; (**b**) Effective refractive indices of graphene SPP and PWG modes.

We plot Figure 1 to demonstrate the presence of Fano resonance. Figure 1a shows the structure we analyzed. Obviously, it’s a typical Otto geometry. To overcome the momentum mismatch with smaller incident angles, we choose Si glass as the coupling prism for its high refractive index (*n_p_* = 3.41) at terahertz frequency. Graphene is marked by a thick red line.

A small air gap lays between the graphene and the coupling prism. Below the graphene, it’s a sensing structure formed by three layered PWG. The graphene is proposed to excite SPP mode, and the waveguide mode is supported by PWG. The structure we proposed makes it possible to couple SPP modes with PWG modes.

The PWG core *n_3_* is selected to be crystal quartz with *n_3_* = 2.1. Cladding layer *n*_2_ is set to be polymethylpentene (TPX) with *n_2_* = 1.46. We assumed the substrate to be water which is the sensing medium and follows a triple Debye function [28,29]:

(1) $$\varepsilon_{water}(\omega )=\frac{\Delta \varepsilon_{1}}{1+j\omega \tau_{1}}+\frac{\Delta \varepsilon_{2}}{1+j\omega \tau_{2}}+\frac{\Delta \varepsilon_{3}}{1+j\omega \tau_{3}}+\varepsilon_{\infty }$$

the Debye-type relaxation strengths in this formulation respectively are $\Delta \varepsilon_{1}=69.1$, $\Delta \varepsilon_{2}=2.01$ $\Delta \varepsilon_{3}=2.08$ and relaxation times $\tau_{1}=9.02\;\mathrm{ps}$, $\tau_{2}=0.80\;\mathrm{ps}$ and $\tau_{3}=0.05\;\mathrm{ps}$. The last term $\varepsilon_{\infty }=2.10$ is the higher frequency limit in the real part. Obviously, the dielectric constant $\varepsilon_{\mathrm{water}}$ is a function of the incident light frequency. In order to get the fixed dielectric constant of water, we assumed the incident light to be transverse magnetic (TM) polarized with a wavelength of 100 μm. We calculated the $\varepsilon_{\mathrm{water}}=3.2127.$ The thickness of each layer is set to be d_1_ = 10 μm, d_2_ = 50 μm and d_3_ = 100 μm, respectively.

Graphene is modeled as a surface conducting sheet and the surface conductivity of grapheme σ is the sum of the intraband $\sigma_{\mathrm{intra}}$ and the interband $\sigma_{\mathrm{inter}}$, which can be expressed by the Kubo formula [30,31]:

(2) $$\sigma_{\mathrm{intra}}=i\frac{e^{2}K_{B}T}{\pi \hbar^{2}(\omega +i\tau )}[\frac{E_{F}}{K_{B}T}+2\ln(e^{-\frac{E_{F}}{K_{B}T}}+1)]$$

(3) $$\sigma_{\mathrm{inter}}=i\frac{e^{2}}{4\pi \hbar }\ln\left|\frac{2E_{F}-(\omega +i\tau )\hbar }{2E_{F}+(\omega +i\tau )\hbar }\right|$$

*E_F_* is determined by carrier density $E_{F}=\hbar V_{F}\sqrt{\pi n}$, The carrier density is controlled by an applied electrostatic potential. $n=C_{g}\Delta V/e$, where $C_{g}=\varepsilon /d$ is the gate capacitance per unit area. *d* and *ɛ* are the distance and electrostatic permittivity of the two electrodes and medium between the two electrodes respectively. Therefore Fermi energy can be dynamically tuned by gate voltage, where $\upsilon_{F}=10^{6}m/s$, *e* is elementary, *ħ* is the reduced Planck’s constant, *K_B_* is the Boltzmann constant. *τ* is the phenomenological relaxation time which is assumed to be 1 ps, the temperature *T* = 300 K. And the effective thickness of monolayer graphene is usually taken to be *d*_g_ = 0.34 nm.

In numerical calculations, we choose the n_p_-n_1_-FLG-n_2_ which consists of prism, air gap, few layer graphene and cladding layer of PWG as a system to match the boundary conditions. The SPP dispersion can be derived as:

(4) $$\tanh\alpha_{1}d_{1}=-\frac{\alpha_{1}\varepsilon_{p}/(\varepsilon_{1}\alpha_{p})+\left(\alpha_{1}\varepsilon_{2}\right)/\left(\alpha_{2}\varepsilon_{1}\right)[1+i\sigma \alpha_{2}/\left(\omega \varepsilon_{0}\varepsilon_{2})\right]}{1+\alpha_{1}{}^{2}\varepsilon_{2}\varepsilon_{p}/\varepsilon_{1}{}^{2}\alpha_{p}\alpha_{2}[1+i\sigma \alpha_{2}/\omega \varepsilon_{0}\varepsilon_{2}]}$$

where $\alpha_{j}=\sqrt{\beta^{2}-k_{0}^{2}\varepsilon_{j}}$, *j* = *p*, *s*, 1, 2, 3, 4, *β* is the *x* component wavenumber, $\varepsilon_{j}$ is the permittivity. For the multilayer graphene, what need to be note is that σ should be replaced by Nσ.

As for the three layer waveguide structure, the dispersion of the PWG mode can be derived as:

(5) $$\tan k_{3z}d_{3}=\frac{k_{3z}(p_{2}\alpha_{2}+p_{s}\alpha_{s})}{k_{3z}^{2}-p_{2}\alpha_{2}p_{s}\alpha_{s}}$$

where $p_{j}=\varepsilon_{3}/\varepsilon_{j}$, $k_{3z}=\sqrt{k_{0}^{2}\varepsilon_{3}-\beta^{2}}$, the two dispersion Equations (4) and (5) are all transcendent equations and can only have a numerical solution. In order to excite the Fano resonance, the effective refractive index ($n_{eff}=\beta /k_{0}$) of graphene SPP and PWG mode should be matched. In Figure 1b, we plot the real part of refractive index according to Equations (4) and (5).

The black solid line is the effective refractive index of the PWG mode which is fixed. The red, blue, and green dashed line are the effective refractive indexes of monolayer graphene SPP, two layer graphene and three layer graphene, respectively. Obviously, they all can make the effective refractive index of graphene SPP and the PWG mode match and excite the Fano resonance. Monolayer graphene can only support SPP with very high Fermi energy. Although Fermi energy of 1 eV has been achieved experimentally [32,33], lower Fermi energies are easier to realize. To reduce the Fermi energy and maintain the effective refractive index at the same time, the layer number of the graphene should be increased. In Figure 1b, we find that when the layer number of the graphene *N* = 3, the corresponding *E_F_* is small enough, so in the following analysis, the layer number of the graphene is considered to be *N* = 3.

The transfer matrix method for the *N*-layer model is used to solve the multilayer systems, shown in Figure 1a. To excite FLG SPP, TM polarized incident light with incident angle is required, the prism and substrate are treated as semi-infinite layers. All layers are assumed to be stacked along in the *z*-direction. The tangential fields at the first boundary *Z* = *Z*_1_ = 0 are related to those at the final boundary *Z* = *Z_N_*_−1_ as [34]:

(6) $$\left[\frac{U_{1}}{V_{1}}\right]=M\left[\frac{U_{N-1}}{V_{N-1}}\right],$$

where *U*_1_ and *V*_1_ are the tangential components of electric and magnetic fields at the boundary of first layer respectively. *U_N_*_−1_ and *V_N_*_−1_ are corresponding fields at the boundary of *N*th layer. *M* is known as the characteristic matrix of the combined structure.

## 3. Results and Discussion

**Figure 2:**
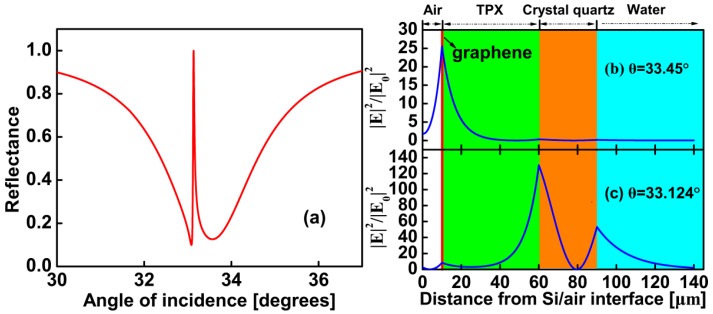
(**a**) ATR spectra calculated as a function of the incident angle at the *E_F_* = 0.37 eV. (**b**) Tangential electric field profiles (normalized by the incident light) for incident angles of 33.450°, (**c**) and for incident angles of 33.124°.

It is known that *E_F_* = 0.37 eV of FLG is appropriate in Figure 1b. We then get the angular reflection spectra of the structure proposed in Figure 1a using the transfer matrix method. As shown in Figure 2a, red solid line shows an attenuated total reflection curve calculated as a function of $\theta_{in}$. The two reflection dips show that both FLG SPP and PWG modes can be excited. FLG SPP is excited around 33.45° which shows a broad reflection dip. PWG mode is excited around 33.09° which shows a much narrower dip. We can see an extremely sharp peak appear between the two modes produced reflection dip in Figure 2a.

In order to verify the two modes have been coupled together and further understand the origin of sharp Fano resonance, we plot Figure 2b,c to show the tangential electric field distributions for the structure. In the figures, the electric field enhancement factor defined as the ratio of the square of the amplitude of the electric field to the square of the incident light. We plot it as a function of the distance *z* which is the distance from interface of Si prism and air. The related reflection spectrum is shown in Figure 2a. In Figure 2b, the incident angle is 33.450°, a strong electric field is generated at the interface of air and FLG and decays exponentially away from the interface. It shows that the electric field distributes mainly in the FLG layer. Figure 2c is obtained with $\theta_{in}=33.124{}^{\circ}$, which corresponds to the middle of the Fano-type resonance in Figure 2a. The strong electric field is not only generated at the interface of air and FLG, but also generated at the PWG region. It shows that the electric field distributes by both FLG layer and PWG. The results presented in Figure 2b,c clearly demonstrate the excitation of hybrid modes of the SPP and PWG modes resulting from the coupling which is believed to be the origin of sharp Fano resonance. 

The characteristic line shape of Fano resonances make them have potential applications in highly sensitive sensors. The change in the refractive index (*n_s_*) of the sensing medium would lead to a change in the angle-scan ATR resonance curve. Corresponding to the change in refractive index of sensing medium (*dn_s_*), the change of reflection is *dR* (sensing by intensity modulation). Then a sensitivity by intensity is given by:

(7) $$S_{I}(\theta )=\frac{dR(\theta )}{dn_{s}}$$

**Figure 3:**
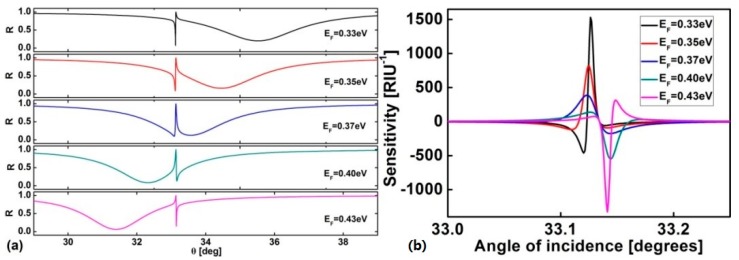
(**a**) Reflection spectra for different values of *E_F_*; (**b**) Different values of *E_F_* corresponding to the sensitivity for the proposed sensor. *E_F_* is taken to be 0.33 eV, 0.35 eV, 0.37 eV, 0.40 eV, 0.43 eV respectively.

The Fermi energy is found to be an important factor affecting the sensitivity. We plot Figure 3 to obtain the effect of *E_F_* on sensitivity. Figure 3a shows the reflectance curves changing with *E_F_* from 0.33–0.43 eV. When *E_F_* = 0.37 eV, the lineshape of the resonance is nearly symmetrical, at this point, the sensitivity is the smallest one in Figure 3b. Whether *E_F_* is increased or decreased, the lineshape of the resonance becomes asymmetrical, and as *E_F_* continues to increase or decrease, the asymmetric lineshape of the reflection becomes sharper, and the sensitivity we calculate is larger. It’s easy to predict that a sharper asymmetric reflection lineshape leads to higher sensitivity.

**Figure 4:**
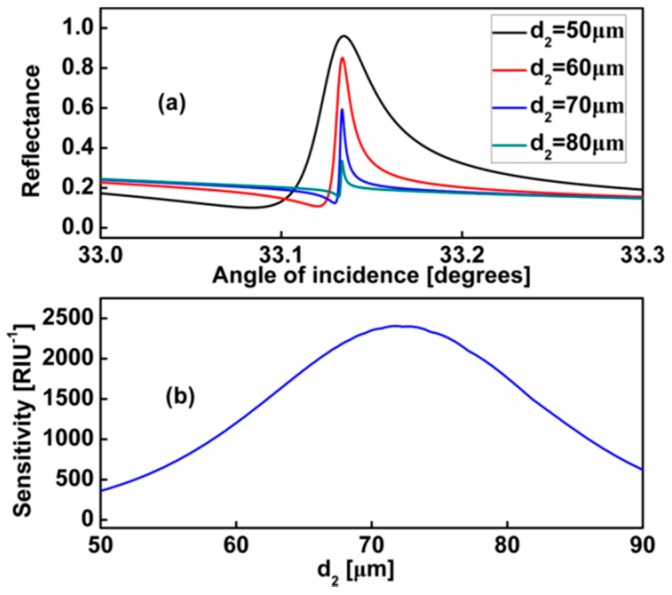
*d*_1_ = 10 μm, *d*_3_ = 30 μm, *E_F_* = 0.37 eV (**a**) Dependence of the Fano lineshape on the thickness of coupling layer *d*_2_; (**b**) Different values *d*_2_ corresponding to the sensitivity for the proposed sensor.

Real waveguide layers produced in laboratories may have losses because of their different physical origins depending on the method and conditions of preparation. A nonzero imaginary part *κ* in the refractive index of the waveguide layer is introduced in our following calculation, which represents the loss of energy in the waveguide layers. We plot Figure 4 to analysis the effect of the coupling layer *d*_2_ on the sensitivity. In Figure 4a, when *d*_2_ increased, the slope of the reflectance curve become steeper. Corresponding to the change of the line shape, the value of the sensitivity becomes larger. We know that PWG mode excited by ATR method have radiation losses due to coupling between PWG and prism [35], and the graphene also has unavoidable intrinsic losses.

When we increase *d*_2_, i.e., the distance between the PWG and the prism or graphene, the radiation loss and intrinsic loss of PWG mode decrease simultaneously, as a natural result the increase of the quality factor of PWG, and a narrower resonance is achieved, but with the resonance sharpened, we also see the degradation of resonance as *d*_2_ increases, i.e., as the coupling strength decreases at the same time. Therefore, we can see the sensitivity increases first and then decreases after getting peak values in Figure 4b.

**Figure 5:**
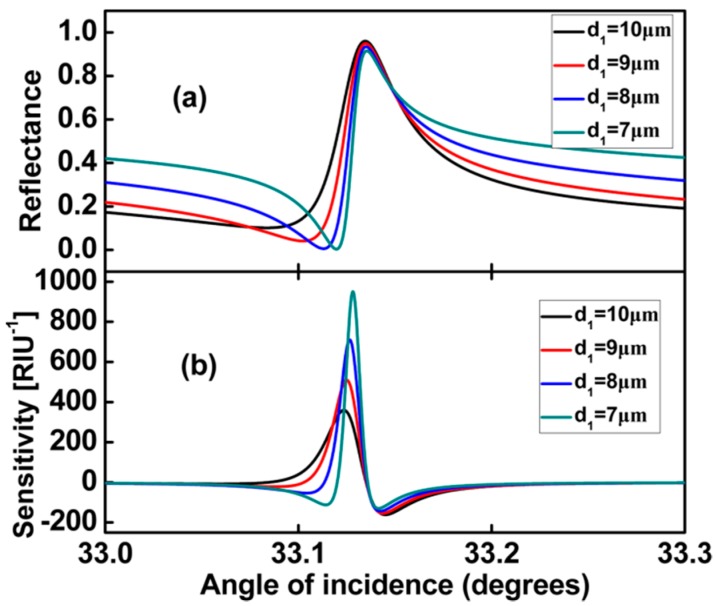
*d*_2_ = 50 μm, *d*_3_ = 30 μm, *E_F_* = 0.37 eV, (**a**) Dependence of the Fano lineshape on the thickness of coupling layer *d*_1_; (**b**) Different values *d*_1_ corresponding to the sensitivity for the proposed sensor.

We plot Figure 5 to analyse the effect of the air layer *d*_1_ on the sensitivity. As *d*_1_ decreases, similarly radiation loss increases a little. The quality factor may become smaller, and the sensitivity is expected to decrease, but we note that when the air layer *d*_1_ is changed, the coupling state changes due to the varying dispersion of graphene SPPs. The lineshape becomes more asymmetric. Comparing the two effects, from Figure 5b, we find that the asymmetric line shape effect is obviously more pronounced.

**Figure 6:**
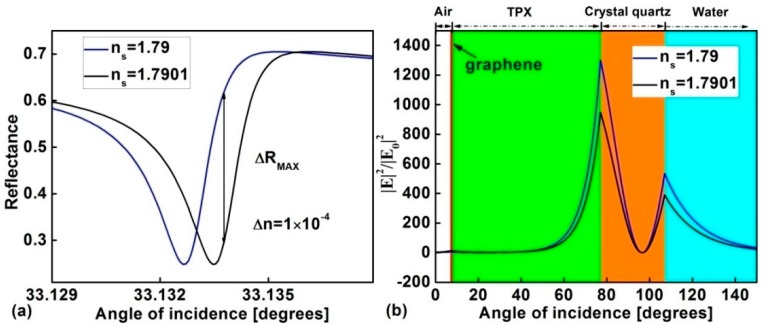
(**a**) Shift of Fano lineshape for the structure with *d*_1_ = 7 μm, *d*_2_ = 70 μm, *E_F_* = 0.35 eV; (**b**) Schematic diagram of the electric field distributions for the proposed Fano resonance sensor at θ=33.1332°.

We plot Figure 6a to show the changes in the ATR spectra for the Fano sensor. We assumed the refractive index is increased by $\Delta n_{s}=1.0\times 10^{-4}$. The change in the reflectance of $\Delta R_{\max}\approx 0.326$. The ratio $\Delta R_{\max}/\Delta n\approx 3260\;{\mathrm{RIU}}^{-1}$. In order to clarify the high sensitivity mechanism of the proposed sensor, corresponding to the change in the *n_s_* in Figure 6a, we calculate the tangential electric field in Figure 6b.

The electric field at the interface of crystal quartz/sensing medium will have an obvious change when *n_s_* has a slight variation. This means that the proposed biosensor is very sensitive to the changes in the sensing medium. 

**Figure 7:**
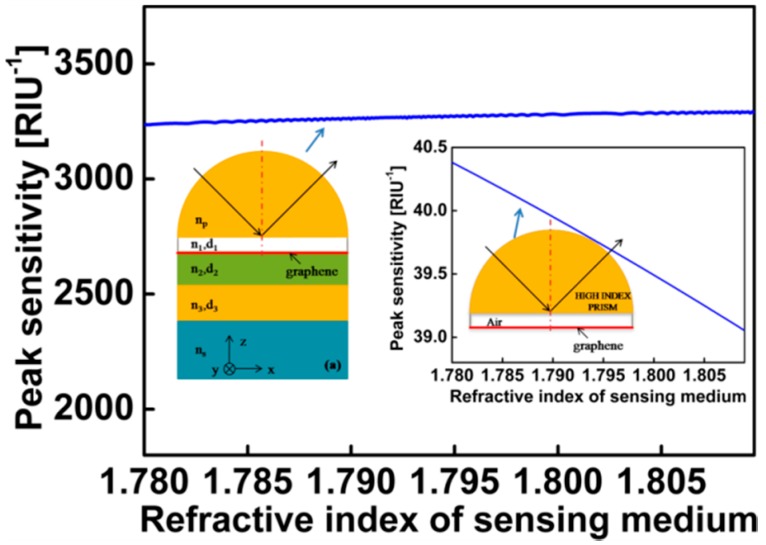
Variation of peak sensitivity with respect to refractive index of sensing medium in the vicinity of water.

We also study the sensitivity changes near the refractive index of water. Figure 7 shows the peak sensitivity calculated as a function of *n_s_*. We can get that the sensitivity is improved as the refractive index of the sensing medium is increased. We compared our sensor with the SPP sensor excited by three layer graphene with *E_F_* = 1 eV and *d*_air_ = 7 μm, which shows that the sensitivities of our proposed structure is two orders of magnitude larger than that with the conventional surface plasmon resonance sensor.

## 4. Conclusions

In this study, we have proposed a terahertz biosensor based on Fano resonance of a graphene/waveguide hybrid structure. The asymmetric lineshape of the Fano resonance is found to have a significant effect on the sensitivity. The sharper the asymmetric line shape of the Fano resonance is, the higher the achieved sensitivity of the biosensor is. The graphene intrinsic losses and radiation losses related to the distance between prism or FLG and the PWG influence the sensitivity a lot. Through the manipulation of structural parameters, we can get sensitivities as high as 3260 RIU^−1^. We believe that the biosensor we proposed will find good use in terahertz wavelength range detection.
